# Pan-Immune Inflammation Value and Clinical Outcomes in Subacute Sclerosing Panencephalitis: A Retrospective Study

**DOI:** 10.3390/v18010018

**Published:** 2025-12-22

**Authors:** Bilge Özgör, Murat Çağlar Şahin, Işınsu Bıçakcıoğlu, Gül Yücel, Meral Karadağ, Serdal Güngör

**Affiliations:** 1 Division of Pediatric Neurology, Department of Pediatrics, Faculty of Medicine, İnönü University, Malatya 44050, Türkiye; drisinsu@gmail.com (I.B.); drgulyucel@hotmail.com (G.Y.); 2 Department of Pediatrics, Faculty of Medicine, İnönü University, Malatya 44050, Türkiye; muratcaglarsahin@gmail.com; 3 Pediatric Neurology Clinic, Malatya Training and Research Hospital, Malatya 44050, Türkiye; mrlkaradag19@gmail.com; 4 Department of Pediatric Neurology, Antalya Medical Park Hospital, Antalya 07010, Türkiye; gungorserdal@yahoo.com

**Keywords:** subacute sclerosing panencephalitis, measles virus, pan-immune inflammation value, prognosis, inflammatory biomarker

## Abstract

Subacute sclerosing panencephalitis (SSPE) is a rare, progressive, and fatal neurological disorder caused by persistent measles virus infection. Reliable prognostic biomarkers remain limited. Systemic inflammation has been implicated in the pathogenesis of neuroinfectious diseases, and hematology-derived indices are increasingly recognized as accessible markers of inflammatory burden. This retrospective case–control study was conducted at İnönü University Faculty of Medicine, Malatya, Türkiye, between 2010 and 2025, including 40 pediatric patients with SSPE and 40 age- and sex-matched healthy controls. Demographic and laboratory data were retrieved from institutional records, and disease severity was classified according to Jabbour stages. Compared with controls, patients with SSPE had significantly higher pan-immune inflammation value (PIV: 710.5 [320–1050] vs. 280.0 [150–460], *p* < 0.001), systemic immune-inflammation index (SII: 640.0 [310–1240] vs. 410.0 [210–720], *p* = 0.02), and neutrophil-to-lymphocyte ratio (NLR: 2.1 [1.2–3.8] vs. 1.6 [1.0–2.5], *p* = 0.03), along with lower lymphocyte counts (*p* = 0.04). Elevated PIVs were strongly associated with advanced Jabbour stages, impaired ambulation, and a higher case-fatality ratio (35%). Multivariate regression identified PIV as an independent predictor of death (OR: 3.25, 95% CI: 1.45–7.28, *p* = 0.004), and receiver operating characteristic analysis demonstrated superior discriminative accuracy of PIV (AUC = 0.87) compared with other indices. These findings suggest that PIV, a simple and inexpensive biomarker derived from routine blood tests, may provide useful prognostic information in SSPE and aid early risk stratification. Further multicenter, prospective studies are warranted to validate its clinical utility.

## 1. Introduction

Subacute sclerosing panencephalitis (SSPE) is a rare, progressive, and invariably fatal neurodegenerative disorder caused by persistent infection with a defective measles virus. First described by Dawson in the early 20th century, SSPE predominantly affects children and adolescents, typically manifesting years after the initial measles infection [1,2]. The global incidence is estimated to range between 4 and 11 cases per 100,000 to 1,000,000 measles infections, with a markedly higher burden reported in developing countries where measles vaccination coverage remains suboptimal [3,4,5]. The disease usually follows a prolonged latent period, after which patients present with cognitive decline, behavioral changes, myoclonus, motor dysfunction, and progressive neurological deterioration, ultimately leading to death within a few years [6].

In Türkiye, measles incidence has shown periodic fluctuations over the last 15 years (2010–2025), with several outbreaks reported between 2013 and 2019 despite nationwide MMR vaccination programs achieving 94–96% coverage. Sporadic clusters have continued through 2023–2024, reflecting regional differences in vaccination uptake and population mobility. Consequently, SSPE remains a clinically relevant condition even in the post-elimination era, particularly among children infected before full immunization coverage was achieved.

The diagnosis of SSPE relies on a combination of clinical features, characteristic periodic electroencephalographic (EEG) discharges, neuroimaging findings, and demonstration of elevated measles-specific antibodies in cerebrospinal fluid (CSF) [6,7]. Among clinical tools, the Jabbour staging system is widely used to grade the severity of neurological impairment and disease progression, ranging from subtle behavioral alterations (Stage I) to severe motor dysfunction and vegetative state (Stage IV) [8,9,10]. Additionally, the diagnosis is commonly established using Dyken’s diagnostic criteria, which integrate progressive cognitive decline, characteristic EEG findings, and high CSF measles antibody titers. Nevertheless, the clinical course of SSPE is highly heterogeneous, and prognostic variability is observed even among patients within the same disease stage. Therefore, there is an urgent need for reliable and easily applicable biomarkers that can predict disease severity and mortality risk.

In recent years, increasing attention has been directed toward the role of systemic inflammation in the pathophysiology of neuroinfectious and neurodegenerative diseases. Dysregulation of the immune response, including chronic activation of innate immunity, has been suggested as a major contributor to SSPE progression [11]. Peripheral blood–derived inflammatory indices have emerged as surrogate markers of systemic immune activation. Among these, the neutrophil-to-lymphocyte ratio (NLR), platelet-to-lymphocyte ratio (PLR), and the systemic immune-inflammation index (SII) have been widely investigated as prognostic indicators in various infectious, neurological, and malignant disorders [12,13,14].

More recently, the pan-immune-inflammation value (PIV) has been introduced as a novel composite biomarker that integrates neutrophil, monocyte, platelet, and lymphocyte counts into a single parameter [15,16]. By capturing multiple cellular compartments of the immune response, PIV provides a more comprehensive reflection of systemic inflammation than conventional indices. Several studies have demonstrated its prognostic value in oncological, cardiovascular, and chronic inflammatory conditions [17,18]. However, no previous research has evaluated its potential role in SSPE, a disease where chronic viral persistence and immune dysregulation coexist.

From a virological perspective, the association between elevated PIV and poor clinical outcomes in SSPE is biologically plausible. Persistent measles virus infection within the central nervous system triggers chronic activation of microglia and astrocytes, resulting in sustained release of proinflammatory cytokines such as IL-6, TNF-α, and IFN-γ. These mediators promote peripheral myelopoiesis, neutrophil and monocyte mobilization, and platelet activation—components that directly elevate PIV. In parallel, the progressive depletion and functional exhaustion of lymphocytes, a hallmark of chronic measles infection, disproportionately amplifies PIV by reducing the denominator of the index. Together, this constellation of innate immune activation, impaired adaptive immunity, and virus-induced neuroinflammatory cascades provides a plausible mechanistic explanation for why PIV mirrors SSPE progression and correlates with neurodegeneration, ambulatory decline, and mortality.

The present study aimed to investigate the association between PIV and clinical outcomes in SSPE, particularly focusing on disease severity and case-fatality ratio. By identifying PIV as a simple, cost-effective, and universally accessible biomarker, this study seeks to contribute to early risk stratification and improved prognostic assessment in this devastating disorder.

## 2. Materials and Methods

### 2.1. Study Design and Setting

This study was designed as a retrospective case–control investigation conducted at the Department of Pediatric Neurology, İnönü University Faculty of Medicine. The department serves as a tertiary referral center for Malatya and neighboring provinces in Eastern Türkiye, covering an estimated population of approximately 2.5 million. Patient data were obtained from institutional medical records and electronic databases covering the period between January 2010 and March 2025. The study was conducted in accordance with the principles of the Declaration of Helsinki and received approval from the Non-Interventional Clinical Research Ethics Committee of İnönü University (Decision No: 2025/8464; Date: 30 September 2025), and all methodological and reporting procedures were performed in compliance with the STROBE guidelines for observational studies.

### 2.2. Study Population

The study included a total of 80 participants, comprising 40 patients diagnosed with subacute sclerosing panencephalitis (SSPE) and 40 age- and sex-matched healthy controls. The diagnosis of SSPE was established according to Dyken’s diagnostic criteria, which include (1) progressive subacute mental deterioration with myoclonus, (2) characteristic periodic EEG discharges, (3) elevated cerebrospinal fluid (CSF) measles antibody titers, and (4) compatible neuroimaging findings [8,9,10]. Patients with SSPE were identified through retrospective review of hospital records. Healthy controls were randomly selected from children who attended the same hospital for routine health examinations and were confirmed to be free of neurological or systemic inflammatory diseases. This selection method ensured that the controls were derived from the same source population as the cases, thereby minimizing selection bias. To ensure data reliability, only participants with complete clinical and laboratory information were included in the analysis.

Exclusion criteria consisted of coexisting chronic systemic illnesses such as autoimmune disease, malignancy, or chronic infection; history of acute infection within the preceding four weeks; incomplete laboratory or clinical documentation; and prior use of corticosteroids, immunosuppressive drugs, or any treatment that could potentially alter systemic inflammatory markers within the last three months.

### 2.3. Data Collection

Demographic variables (age and sex) and clinical information (disease duration, Jabbour stage, ambulatory status, and survival outcome) were retrieved from patient files. Laboratory results were obtained from the first admission records. Complete blood count (CBC) analyses were performed on venous blood samples using an automated hematology analyzer (Sysmex XN-1000, Sysmex Corporation, Kobe, Japan). Standard biochemical measurements, including glucose and lipid levels when available, were performed using an automated biochemistry analyzer (Roche Cobas 8000, Roche Diagnostics, Basel, Switzerland). All assays were conducted in the central laboratory of İnönü University Hospital, which is accredited for routine clinical testing.

From CBC data, systemic inflammatory indices were calculated, including the neutrophil-to-lymphocyte ratio (NLR), platelet-to-lymphocyte ratio (PLR), systemic immune-inflammation index (SII; platelet × neutrophil/lymphocyte), and pan-immune-inflammation value (PIV; neutrophil × platelet × monocyte/lymphocyte). All calculations were carried out manually and cross-checked by two independent researchers.

### 2.4. Clinical Outcome Measures

The primary clinical outcomes assessed in this study were disease severity, functional status, and case-fatality ratio (CFR). Disease severity was graded using the Jabbour classification, which categorizes SSPE into four stages based on neurological deterioration, ranging from early cognitive and behavioral changes (Stage I) to severe motor dysfunction, unresponsiveness, and vegetative state (Stage IV). Functional mobility was systematically evaluated through ambulation status, which was classified into three categories: independent ambulation, assisted ambulation, and non-ambulatory condition. This classification allowed a clear assessment of the patient’s physical dependence in daily activities. The CFR was defined as the proportion of deceased patients among all SSPE cases during the study period.

### 2.5. Statistical Analysis

Statistical analysis was performed using IBM SPSS Statistics for Windows, Version 22.0 (IBM Corp., Armonk, NY, USA). The normality of continuous variables was assessed with the Shapiro–Wilk test. Variables with normal distribution were presented as mean ± standard deviation, while non-normally distributed variables were presented as median (minimum–maximum). Comparisons between two groups were performed using the independent samples t-test for normally distributed data and the Mann–Whitney U test for non-normally distributed data. Categorical variables were expressed as frequency (percentage) and compared using the chi-square test or Fisher’s exact test when expected frequencies were low.

Univariate logistic regression analysis was conducted to evaluate the association of inflammatory indices (PIV, SII, NLR, PLR) with disease severity and CFR. Variables with statistical significance in univariate analysis were entered into a multivariate logistic regression model. Results were expressed as odds ratios (OR) with 95% confidence intervals (CI). To assess the predictive accuracy for fatal outcomes, receiver operating characteristic (ROC) curve analysis was performed and the area under the curve (AUC) was calculated. The optimal cutoff value was determined using the Youden index. A *p*-value < 0.05 was considered statistically significant.

Sample size estimation was performed using G*Power 3.1. Based on an expected case-fatality rate of 35% and assuming a moderate effect size (OR ≈ 1.8) with α = 0.05, at least 72 participants were required to achieve 80% statistical power. The present study included 80 participants (40 SSPE cases and 40 controls), thereby exceeding the required sample size and ensuring sufficient statistical power [7].

## 3. Results

According to Table 1, the mean age of the SSPE group was 10.2 ± 3.1 years, comparable to 9.8 ± 2.9 years in the control group (*p* = 0.56). Male predominance was similar between groups (60.0% vs. 55.0%, *p* = 0.64). The median disease duration among SSPE patients was 2.5 years [IQR: 1.0–4.0]. Disease staging revealed that 40.0% of patients were in early phases (Stages I–II), whereas 60.0% were in advanced phases (Stages III–IV). At evaluation, 30.0% of patients were independently ambulatory, 40.0% required assistance, and 30.0% were non-ambulatory. The case-fatality ratio (CFR) was 35.0% (14/40).

Additionally, 6 patients (15.0%) had received recent immunomodulatory treatment (corticosteroids, IVIG, or antiviral therapy) within the preceding four weeks. Documented causes of death included progressive neurological deterioration (57.1%), aspiration pneumonia (28.6%), and cachexia/malnutrition (14.3%). These newly added clinical variables are summarized in Table 1.

According to Table 2, white blood cell and neutrophil counts were slightly higher in SSPE patients (7.0 ± 2.1 vs. 6.6 ± 1.8 × 10^3^/µL and 3.9 ± 1.2 vs. 3.5 ± 1.0 × 10^3^/µL), though differences were not significant (*p* = 0.28 and 0.09, respectively). Lymphocyte counts were significantly lower in the SSPE group (2.1 ± 0.7 vs. 2.4 ± 0.6 × 103/µL, *p* = 0.04), while platelet and monocyte counts were comparable (*p* = 0.07 and 0.08). Among hematologic indices, median PIV was 710.5 [320–1050] in SSPE versus 280.0 [150–460] in controls (*p* < 0.001). SII was 640.0 [310–1240] versus 410.0 [210–720] (*p* = 0.02), and NLR 2.1 [1.2–3.8] versus 1.6 [1.0–2.5] (*p* = 0.03). PLR showed no significant difference (150 [100–290] vs. 130 [90–220], *p* = 0.12).

When stratified by clinical outcomes (Table 3), median PIVs increased progressively with disease severity and loss of ambulation. Patients in Jabbour Stages III–IV had significantly higher PIV levels than those in Stages I–II (980.0 [650–1320] vs. 420.0 [200–620]; *p* < 0.001). Similarly, non-ambulatory patients showed the highest median PIV (1120.0 [760–1450]) compared with ambulatory individuals (410.0 [200–630] and 720.0 [450–950]; *p* = 0.002). Patients who died had markedly elevated PIVs (1240.0 [880–1680]) compared with survivors (520.0 [280–760]; *p* < 0.001).

Multivariate logistic regression analysis (Table 4) demonstrated that elevated PIV was independently associated with fatal outcomes (OR = 3.25, 95% CI = 1.45–7.28, *p* = 0.004) after adjustment for age, sex, and SII. None of the other indices retained statistical significance (*p* > 0.05).

Receiver operating characteristic analysis (Table 5) showed that PIV had the highest diagnostic performance for predicting fatal outcomes (AUC = 0.87, 95% CI = 0.78–0.96, *p* < 0.001), with an optimal cut-off of 880.0, yielding 82% sensitivity and 79% specificity. SII followed with an AUC of 0.75 (*p* = 0.01; cut-off = 700.0; sensitivity 72%; specificity 71%), while NLR (AUC = 0.71; *p* = 0.02) and PLR (AUC = 0.62; *p* = 0.11) demonstrated lower predictive accuracy.

The independence of inflammatory indices included in the regression analyses was assessed by calculating variance inflation factor (VIF) values. As shown in Table 6, none of the variables exhibited VIF values exceeding the commonly accepted threshold of 5 (all *p*-values for model fit > 0.05), indicating that the regression model was not affected by multicollinearity.

A multivariate logistic regression model using continuous inflammatory parameters was constructed to identify independent predictors of fatal outcome. As presented in Table 7, PIV demonstrated a statistically significant association with mortality (*p* = 0.008), whereas age, sex, SII, NLR, PLR, and recent immunomodulatory therapy showed no significant associations (all *p* > 0.05).

## 4. Discussion

This retrospective case–control study investigated the relationship between hematology-derived systemic inflammatory indices and clinical outcomes in patients with subacute sclerosing panencephalitis (SSPE). Our results demonstrated that the pan-immune-inflammation value (PIV) was significantly associated with disease severity and independently predicted mortality. To our knowledge, this is the first study to evaluate the prognostic significance of PIV in SSPE, providing preliminary evidence that this accessible and cost-effective biomarker may have clinical relevance in prognostic assessment.

In our cohort, the mean age and male predominance were consistent with previous studies reporting that SSPE predominantly affects male children and adolescents [19,20]. The relatively high proportion of patients classified as Jabbour stage III–IV indicates that diagnosis often occurs at advanced stages, consistent with reports emphasizing delayed recognition in endemic regions [5,21,22]. The case-fatality ratio (CFR) of 35% observed in this study aligns with prior reports ranging from 25% to 45%, confirming the devastating natural history of SSPE.

From an epidemiological standpoint, the study period (2010–2025) corresponds to a time of fluctuating measles incidence in Türkiye, with intermittent outbreaks following periods of reduced vaccination coverage. Although national immunization programs have significantly decreased measles prevalence, regional heterogeneity in measles–mumps–rubella (MMR) vaccine uptake persists, especially in socioeconomically disadvantaged areas. These variations likely explain the continued occurrence of SSPE in tertiary care centers such as ours.

With regard to hematologic findings, leukocyte and neutrophil counts were slightly higher in the SSPE group but did not reach statistical significance, while lymphocyte counts were significantly lower. This pattern supports the hypothesis that chronic viral infections disrupt immune homeostasis, leading to impaired lymphocyte-mediated immune responses and sustained systemic inflammation [6,23,24]. Monocyte and platelet counts were comparable between groups, yet monocytes may indirectly influence inflammatory burden since they contribute directly to the PIV formula.

Among hematologic indices, the neutrophil-to-lymphocyte ratio (NLR) and systemic immune-inflammation index (SII) were significantly elevated in SSPE patients, in line with previous evidence linking these markers to poor outcomes in infectious and neurodegenerative diseases [25,26,27,28]. However, only PIV retained independent prognostic value in multivariate analysis, suggesting that this composite marker—by integrating multiple cellular elements of the immune system—may better reflect the overall inflammatory milieu than simpler ratios such as NLR or PLR.

The most notable finding of our study was the strong association between elevated PIV and both disease severity and mortality. Patients with higher PIVs were more likely to present with advanced Jabbour stages and impaired ambulation and had a markedly increased risk of death. PIV has been established as a robust prognostic biomarker in oncology, cardiovascular disorders, and sepsis [16,29,30]. Our findings extend this concept to neuroinfectious diseases, suggesting that systemic inflammation—captured by PIV—may mirror the intensity of neuroimmune activation and disease progression in SSPE. The mechanistic basis may involve chronic activation of innate immune pathways, increased production of proinflammatory cytokines, and relative lymphopenia due to immune exhaustion, all of which have been implicated in the pathogenesis of SSPE [6,23].

From a virological perspective, chronic measles virus persistence triggers sustained microglial and astrocytic activation, leading to continuous release of IL-6, TNF-α, and IFN-γ. These cytokines promote peripheral neutrophil and monocyte mobilization and platelet activation, while long-standing measles infection induces lymphocyte exhaustion and depletion. This immunological constellation directly elevates PIV and provides a biologically plausible explanation for its strong correlation with neurodegeneration, ambulatory decline, and mortality in SSPE. However, because all biomarkers in this study were obtained at a single time point, the dynamic inflammatory evolution characteristic of progressive measles persistence could not be captured, and acute intercurrent infections could not be fully differentiated from chronic neuroinflammation.

Beyond these clinical associations, additional methodological analyses were performed to ensure the robustness of the observed relationships. Because composite indices such as PIV may share mathematical components with other hematologic markers, all inflammatory variables included in regression modeling were evaluated for multicollinearity. The absence of elevated VIF values indicated that PIV provided independent information rather than reflecting redundancy with NLR, SII, or PLR. Moreover, an expanded multivariate model incorporating continuous inflammatory parameters and recent immunomodulatory treatments demonstrated that PIV remained the only significant predictor of mortality, suggesting that its prognostic relevance is not confounded by prior antiviral, corticosteroid, or IVIG exposure. Importantly, evaluating inflammatory indices as continuous variables helped mitigate the information loss and potential overfitting associated with dichotomization at Youden-derived cut-offs, reinforcing the stability of PIV as a prognostic marker independent of threshold selection. From a biological standpoint, the integrated components of PIV—neutrophils, monocytes, and platelets in the numerator and lymphocytes in the denominator—may collectively reflect the systemic signature of chronic measles-driven neuroinflammation characteristic of SSPE.

Despite its novelty, this study has several limitations that warrant careful consideration. First, the retrospective and single-center design introduces inherent risks of selection bias and limits the ability to establish causal relationships. Reliance on existing medical records also restricts standardization of clinical assessments and laboratory timing. Second, the modest sample size of 40 SSPE patients reduces the statistical power of subgroup comparisons and limits the precision of effect estimates. Third, all hematologic biomarkers were measured at a single time point, which prevents evaluation of longitudinal inflammatory trajectories and restricts differentiation between chronic neuroinflammatory activity and transient fluctuations due to intercurrent infections. Fourth, although exclusion criteria attempted to minimize confounding, several relevant clinical variables—including nutritional status, subclinical infections, disease duration variability, and detailed information on antiviral therapy, corticosteroid use, or prior IVIG exposure—were not uniformly available and may influence systemic inflammatory indices. Fifth, the study did not incorporate virological parameters such as measles virus genotype, antibody avidity, or CSF PCR results, which could have provided mechanistic context linking hematologic shifts to viral persistence. Additionally, the causes of death were not adjudicated and likely represented a heterogeneous group including brainstem dysfunction, pneumonia, and complications of immobility, introducing variability within the mortality endpoint. Sixth, the absence of comparator groups with other pediatric neuroinflammatory or neurodegenerative disorders—such as autoimmune encephalitis, acute disseminated encephalomyelitis, or viral encephalitides—precludes evaluation of disease specificity; thus, elevated PIV should be interpreted as a general marker of systemic inflammation rather than an SSPE-specific signature. Finally, the findings require cautious interpretation given the lack of external validation, and generalizability remains uncertain without replication in larger, multi-center prospective cohorts incorporating harmonized clinical, immunological, and virological assessments.

## 5. Conclusions

In conclusion, this study provides the first evidence that the pan-immune-inflammation value (PIV), a composite marker derived from routine hematologic parameters, may serve as an independent predictor of disease severity and mortality in patients with subacute sclerosing panencephalitis (SSPE). PIV reflects the integrated activity of multiple immune cell lines and may therefore provide a more comprehensive representation of systemic inflammation than conventional indices. Given its simplicity, reproducibility, and negligible cost, PIV could represent a valuable adjunct in the clinical evaluation and risk stratification of SSPE patients.

However, the retrospective design, limited sample size, and single-center nature of the study necessitate cautious interpretation. Further large-scale, prospective, and multicenter investigations are required to validate these findings, explore dynamic changes in inflammatory indices over the disease course, and elucidate the pathophysiological mechanisms linking systemic inflammation with neurodegeneration in SSPE. Integrating PIV with immunologic and neuroimaging biomarkers in future studies may enhance prognostic modeling and facilitate early identification of patients at high risk for unfavorable outcomes.

## Figures and Tables

**Table 1:** Demographic and Clinical Characteristics of the Study Population.

Characteristics	SSPE Group (*n* = 40)	Control Group (*n* = 40)	*p*-Value
Age (years, mean ± SD)	10.2 ± 3.1	9.8 ± 2.9	0.56
Sex (Male/Female)	24/16	22/18	0.64
Disease duration (years, median [IQR])	2.5 [1.0–4.0]	—	—
Jabbour Stage I	6 (15.0%)	—	—
Jabbour Stage II	10 (25.0%)	—	—
Jabbour Stage III	14 (35.0%)	—	—
Jabbour Stage IV	10 (25.0%)	—	—
Ambulation—Independent	12 (30.0%)	—	—
Ambulation—Assisted	16 (40.0%)	—	—
Ambulation—Non-ambulatory	12 (30.0%)	—	—
Case-Fatality Ratio (CFR)	14/40 (35.0%)	0/40 (0.0%)	<0.001 *
Recent immunomodulatory therapy (steroids/IVIG/antivirals)	6 (15.0%)	—	—
Documented causes of death	Neurological decline: 8 (57.1%)	—	—
	Aspiration pneumonia: 4 (28.6%)	—	—
	Cachexia/malnutrition: 2 (14.3%)	—	—

SD, standard deviation; IQR, interquartile range; SSPE, subacute sclerosing panencephalitis; CFR, Case-Fatality Ratio. * Statistically significant at *p* < 0.05.

**Table 2:** Laboratory Parameters and Inflammatory Indices in SSPE and Control Groups.

Parameters	SSPE Group (*n* = 40)	Control Group (*n* = 40)	*p*-Value
WBC (×103/µL, mean ± SD)	7.0 ± 2.1	6.6 ± 1.8	0.28
Neutrophils (×103/µL)	3.9 ± 1.2	3.5 ± 1.0	0.09
Lymphocytes (×103/µL)	2.1 ± 0.7	2.4 ± 0.6	0.04 *
Monocytes (×103/µL)	0.6 ± 0.2	0.5 ± 0.2	0.08
Platelets (×103/µL)	280 ± 50	300 ± 40	0.07
NLR (median [IQR])	2.1 [1.2–3.8]	1.6 [1.0–2.5]	0.03 *
PLR (median [IQR])	150.0 [100.0–290.0]	130.0 [90.0–220.0]	0.12
SII (median [IQR])	640.0 [310.0–1240.0]	410.0 [210.0–720.0]	0.02 *
PIV (median [IQR])	710.5 [320.0–1050.0]	280.0 [150.0–460.0]	<0.001 *

WBC, white blood cell; NLR, neutrophil-to-lymphocyte ratio; PLR, platelet-to-lymphocyte ratio; SII, systemic immune-inflammation index; PIV, pan-immune inflammation value; SD, standard deviation; IQR, interquartile range; SSPE, subacute sclerosing panencephalitis. * Statistically significant at *p* < 0.05.

**Table 3:** Association Between PIV and Clinical Outcomes in SSPE Patients.

Variables	PIV (Median [IQR])	*p*-Value
Jabbour Stage I–II (n = 16)	420.0 [200.0–620.0]	—
Jabbour Stage III–IV (n = 24)	980.0 [650.0–1320.0]	<0.001 *
Ambulation—Independent	410.0 [200.0–630.0]	—
Ambulation—With assistance	720.0 [450.0–950.0]	—
Ambulation—Non-ambulatory	1120.0 [760.0–1450.0]	0.002 *
Survivors (n = 26)	520.0 [280.0–760.0]	—
Deceased (n = 14)	1240.0 [880.0–1680.0]	<0.001 *

Abbreviations: PIV, pan-immune inflammation value; IQR, interquartile range; SSPE, subacute sclerosing panencephalitis. * Statistically significant at *p* < 0.05.

**Table 4:** Logistic Regression Analysis for Predictors of Fatal Outcome in SSPE Patients.

Variables	OR (95% CI)	*p*-Value
Age	1.08 (0.92–1.26)	0.35
Male sex	1.42 (0.51–3.98)	0.49
SII	1.15 (0.88–1.72)	0.16
NLR	1.20 (0.79–1.98)	0.28
PLR	0.94 (0.62–1.41)	0.74
PIV	3.25 (1.45–7.28)	0.004 *

OR, odds ratio; CI, confidence interval; SII, systemic immune-inflammation index; NLR, neutrophil-to-lymphocyte ratio; PLR, platelet-to-lymphocyte ratio; PIV, pan-immune inflammation value; SSPE, subacute sclerosing panencephalitis. * Statistically significant at *p* < 0.05.

**Table 5:** ROC Curve Analysis of Inflammatory Indices for Predicting Fatal Outcome in SSPE.

Parameters	AUC (95% CI)	Cut-Off	Sensitivity (%)	Specificity (%)	*p*-Value
NLR	0.71 (0.59–0.84)	2.8	68.0	65.0	0.02 *
PLR	0.62 (0.48–0.76)	160.0	58.0	60.0	0.11
SII	0.75 (0.62–0.88)	700.0	72.0	71.0	0.01 *
PIV	0.87 (0.78–0.96)	880.0	82.0	79.0	<0.001 *

AUC, area under the curve; CI, confidence interval; ROC, receiver operating characteristic; NLR, neutrophil-to-lymphocyte ratio; PLR, platelet-to-lymphocyte ratio; SII, systemic immune-inflammation index; PIV, pan-immune inflammation value; SSPE, subacute sclerosing panencephalitis. * Statistically significant at *p* < 0.05.

**Table 6:** Variance inflation factor (VIF) values of inflammatory indices included in regression analyses.

Variable	VIF	Tolerance
PIV (Pan-Immune Inflammation Value)	2.10	0.48
SII (Systemic Immune-Inflammation Index)	2.80	0.36
NLR (Neutrophil-to-Lymphocyte Ratio)	3.00	0.33
PLR (Platelet-to-Lymphocyte Ratio)	1.90	0.52

VIF, variance inflation factor; PIV, pan-immune inflammation value; SII, systemic immune-inflammation index; NLR, neutrophil-to-lymphocyte ratio; PLR, platelet-to-lymphocyte ratio.

**Table 7:** Multivariate logistic regression model evaluating predictors of fatal outcome in SSPE patients using continuous inflammatory parameters.

Variable	Adjusted OR (95% CI)	*p*-Value
Age	1.05 (0.89–1.23)	0.56
Male sex	1.28 (0.45–3.62)	0.64
PIV (continuous)	2.94 (1.31–6.52)	0.008
SII (continuous)	1.09 (0.82–1.55)	0.28
NLR (continuous)	1.14 (0.76–1.81)	0.42
PLR (continuous)	0.92 (0.61–1.38)	0.70
Recent immunomodulatory therapy	1.37 (0.42–4.32)	0.59

OR, odds ratio; CI, confidence interval; SSPE, subacute sclerosing panencephalitis; PIV, pan-immune inflammation value; SII, systemic immune-inflammation index; NLR, neutrophil-to-lymphocyte ratio; PLR, platelet-to-lymphocyte ratio.

## Data Availability

The data presented in this study are available on reasonable request from the corresponding author. The data are not publicly available due to ethical and privacy restrictions.

## Abbreviations

SSPE	Subacute Sclerosing Panencephalitis
PIV	Pan-Immune Inflammation Value
SII	Systemic Immune-Inflammation Index
NLR	Neutrophil-to-Lymphocyte Ratio
PLR	Platelet-to-Lymphocyte Ratio
ROC	Receiver Operating Characteristic
AUC	Area Under the Curve
OR	Odds Ratio
CI	Confidence Interval
WBC	White Blood Cell
Hb	Hemoglobin
CRP	C-Reactive Protein
CBC	Complete Blood Count
CSF	Cerebrospinal Fluid
EEG	Electroencephalography
IQR	Interquartile Range
SD	Standard Deviation
